# The effects of biophilic design on steering performance in virtual reality

**DOI:** 10.1038/s41598-025-19113-4

**Published:** 2025-09-12

**Authors:** Fariba Mostajeran, Moritz Friedrich, Frank Steinicke, Simone Kühn, Wolfgang Stuerzlinger

**Affiliations:** 1 Department of Informatics, Human-Computer Interaction Group, University of Hamburg, Hamburg, 22527 Germany; 2 Clinic and Polyclinic for Psychiatry and Psychotherapy, Neural Plasticity Group, University Medical Center Hamburg-Eppendorf, Hamburg, 20246 Germany; 3 Center for Environmental Neuroscience, Max Planck Institute for Human Development, Berlin, 14195 Germany; 4 School of Interactive Arts and Technology, Simon Fraser University, Surrey, BC V3T 0A3 Canada

**Keywords:** Psychology, Computer science

## Abstract

Biophilic design aims to connect people to nature by incorporating natural elements such as plants in built environments. In immersive virtual reality (VR) environments, experiments have shown that, for instance, the presence of virtual plants in VR leads to higher cognitive performance and psychological well-being. However, it has not been investigated so far whether such effects extend to the performance of spatial interaction tasks such as path steering. In this paper, we explore the effects of the presence of virtual plants in an immersive virtual office environment on path steering performance in VR. For this purpose, we combined and replicated two previous studies in this field. The results of our study did not find an effect of the presence of virtual plants on steering time, as our participants performed all steering tasks in a similar amount of time in both biophilic and non-biophilic environments. We could, however, replicate one of the original studies on path steering in VR by Liu et al. and show that the steering time in our study also follows the extension of the steering law proposed in their work. Accordingly, we found not only a significant effect of the length ($p<.001$, effect size: partial eta squared $=.93$) and width ($p<.001$, effect size =.96) of the steering path, but also a significant effect of the path curvature ($p=.003$, effect size $=.64$) on the time needed for steering through the path. We also found significant effects of path latitude ($p<.001$, effect size $=.57$) and longitude ($p=.01$, effect size $=.18$) on steering time. Our findings challenge the previous research, which demonstrated the positive effects of biophilic design in VR on cognitive processes, and therefore, pave the path for future work to better understand the effects of biophilic design on users’ performance in VR.

## Introduction

Biophilic design promotes engagement in natural features and processes in various settings, including interior, exterior, and transitional spaces and landscapes^1^. It provides different design strategies for incorporating natural elements such as natural materials, plants, views, and vistas into the indoor environment. Other basic elements of a biophilic design include natural shapes, forms, patterns, and lighting. Additionally, place-based relationships such as the geographic connection to a place and evolved human-nature relationships can be observed in such designs^2^.

Biophilic design is an effort to connect people more to nature in the era of urbanization, where more than half of the world’s population lives in cities and spends most of their time in artificially designed living and working spaces^3,4^. Many hours spent teleworking, which spread during the COVID-19 pandemic and could increase in the future with new immersive workspaces and broader utilization of the metaverse, will continue restricting people from accessing and connecting with nature regularly^5–8^. The idea behind biophilic design is the biophilia hypothesis, which suggests that the desire to connect to nature is ingrained in human biology^9^. Numerous studies have empirically confirmed the positive effects of exposure to and interaction with nature on human psychology, physiology, and cognition^10–13^. For instance, it has been shown that natural environments activate the human parasympathetic nervous system in a way that reduces stress^14,15^.

Similar efforts have been made to study the effects of simulations of nature on human psychology and physiology^16^. These simulations span a wide variety of characteristics, including their content and the mode of presentation to users^17,18^. Nonetheless, research has demonstrated that even simple photographs of nature elicit psychological responses that are similar to responses to actual natural environments^19^. Studies have also made use of immersive virtual reality (VR) technologies to bring users closer to nature^20,21^. An advantage of using immersive VR for this purpose is that the immersive properties of VR head-mounted displays (HMDs) facilitate providing the illusion of being physically present in the displayed virtual nature^22,23^. Multiple studies have compared exposure to immersive VR versus real nature exposures and have demonstrated that virtual nature can induce similar positive effects as real nature^24–28^.

Previous research has also studied the effects of immersive biophilic designs on the psycho- and physiological responses of the users^29,30^. For instance, Yin et al. observed a 14% improvement in short-term memory due to exposure to a biophilic office in VR^28^. However, there is a gap in the literature when it comes to the effects of biophilic design on spatial interaction tasks in VR. A reason might be that the previous studies have mostly used VR merely as a tool to display biophilic or non-biophilic content without reporting on interactivity with the VR content. The measures employed also assess the psycho-physiological and cognitive responses of the users. For reporting the effects of virtual nature and biophilic design on cognitive processes, previous studies have included cognitive tests to measure working memory, attention, creativity, and overall cognitive functioning^31^.

We showed in our previous work^32^ that the presence of virtual plants in an immersive VR office environment can lead to improvements in both divergent and convergent cognitive tasks and higher psychological well-being. Additionally, research suggests that cognitive and motor processes may be functionally related^33^. Thus, a question arises whether the presence of virtual plants could also be beneficial for motor-cognitive processes, such as a steering task. Since this research question has not been studied before, we set out to examine the potential of virtual plants for this purpose and to explore whether the positive effects of nature on cognitive performance extend to spatial interaction tasks in VR. To address this basic but practically relevant research question, we conducted a VR experiment to study the effects of virtual nature on a standard 3D interaction task. To do so, we combined two previous studies: (i) we used the same virtual office environments as in our previous study^32^, and to study whether our results extend to a motor-cognitive task, (ii) we utilized the steering tasks presented by Liu et al.^34^ in the virtual office with or without virtual plants. The proposed steering tasks by Liu et al. extend the original steering law and account for the path curvature, in addition to path length and width. Therefore, we hypothesized that, similar to their study, **(H1)** the steering time in our study would follow the extension of the steering law. In addition, we hypothesized that, similar to the results of our previous study^32^, **(H2)** the presence of virtual plants would lead to better steering performance, measurable in terms of lower steering time in the virtual office with plants compared to the one without plants. We also decided to additionally measure the simulator sickness, sense of presence, and perceived restorativeness for each virtual environment (i.e., virtual office with or without virtual plants). Although, we did not measure simulator sickness after exposure to each virtual environment in our previous study, but since we found a positive effect of virtual plants on all our other measures for psychological well-being, we hypothesized that in this study, we could also observe **(H3)** a lower simulator sickness in the virtual office with plants compared to the virtual office without plants. Finally and based on our previous findings, we hypothesized that **(H4)** the presence of the virtual plants would also lead to a higher sense of presence and **(H5)** higher perceived restorativeness of the virtual environment. All in all, the contributions of this paper include an empirical evaluation of the effects of virtual biophilic design on steering tasks in VR and a discussion of the results in the context of VR design.

## Related work

### Nature and biophilic design

A vast body of research has provided evidence for the positive effects of nature on human mental and physical health^35–39^. For instance, it has been shown that exposure to nature results in reductions of anxiety^40^ and improved psychological well-being^41^. These effects have also been measured in physiological reactions such as reduced blood pressure^14^ and the stress-related cortisol hormone^42^.

The Biophilia hypothesis proposes that the positive effects of nature arise from an inherent biological connection between humans and the natural environment^9^. This concept has informed the development of two theories within the field of Environmental Psychology. The first theory, known as Stress Reduction Theory (SRT)^14^, claims that natural settings facilitate stress recovery by regulating physiological arousal, increasing positive emotions, and diminishing negative or stress-related feelings^14,15^. These factors contribute to improved psychological well-being through interactions with and exposure to natural environments. The second theory revolves around the influence of nature on attentional capacities. In cognitive psychology, directed attention (also known as voluntary attention) is the ability to focus on a task that is not particularly interesting and therefore requires effort to concentrate^43^. This ability is finite and is inevitably associated with attention fatigue with increased effort^10^. This condition is also referred to as mental fatigue in those affected and is characterized by an increase in performance errors and increased irritability. According to Attention Restoration Theory (ART)^44^, natural environments are particularly suitable for restoring directed attention as they contain stimuli that gently attract involuntary attention (i.e., attention that requires no effort, e.g., paying attention to an interesting event), with the result that directed attention is replenished^44^.

Based on these theories, biophilic design suggests that built environments can promote restorative effects by integrating natural elements into their design^45^. There are a lot of possibilities for incorporating biophilic design elements into buildings. Some of the most frequently employed strategies are installing images of natural scenes and providing sights of plants or views of nature (e.g., through a window). But there are also other ways, such as using natural materials, textures, colors, and nature-inspired shapes, forms, and geometries^2^.

The positive effects of biophilic design have been demonstrated by several studies in real-world settings. For instance, Ulrich et al.^46^ explored how a view of a biophilic garden influenced aggressive behavior in patients in a psychiatric ward. Their results revealed that patients who could see the biophilic garden displayed less aggressive behavior than those who did not have such a view. Furthermore, Dijkstra et al.^47^ studied the effects of indoor plants on stress reduction in hospital rooms. Their findings indicated that the presence of indoor plants significantly lowered self-reported stress levels among patients and enhanced their mood and sense of comfort. In the work context, Nieuwenhuis et al^48^. found that biophilic workspaces (i.e., enriched with plants) significantly increase workplace satisfaction, self-reported levels of concentration, perceived air quality, and objective measures of productivity compared to non-biophilic working spaces. These improvements were also sustained over both their short-term and long-term studies. In a review of 45 papers, Hung et al.^49^ concluded that biophilic design results in positive emotions, attention restoration, relaxation, reduction of anger, and increased cognitive functionality and performance.

### Biophilic design in VR

Previous studies have also examined the effects of biophilic design in VR, but have only partially confirmed their positive effects in line with SRT and ART. For example, Jun et al.^50^ demonstrated that biophilic design (view of nature or a plant wall) in a virtual hospital room can be beneficial for the neurophysiological responses of the participants as it reduces tension (measured using EEG). Their subjective measures also showed that the biophilic design induced positive emotional changes and reduced negative emotions. In another study, Emamjomeh et al.^51^ found that biophilic design (window view to nature) reduced negative affect in both VR and real indoor environments. However, exposure to only their real biophilic environment could improve visual working memory, stress level tests, and positive affect.

Yin et al.^28–30^ performed a series of studies to examine the effects of biophilic office designs in VR. In their first study^28^, participants were physically or virtually (through an immersive video of the same physical environment in VR) exposed to an indoor environment with or without plants. They observed that systolic blood pressure was lower in both real and virtual biophilic environments compared to non-biophilic ones. However, diastolic blood pressure and skin conductance levels were only lower in the virtual biophilic environment compared to a non-biophilic environment. These measures were not significantly different between biophilic and non-biophilic real environments. They also observed higher cognitive performance in the biophilic real environment compared to a non-biophilic one. In their follow-up studies, Yin et al.^29,30^ used computer-generated office environments in VR. Similar to an immersive video of a biophilic indoor environment, physiological indicators of stress, such as skin conductance level, showed consistently lower levels compared to non-biophilic virtual environments. In their 2019 study^29^, the biophilic design had positive effects on creativity but a negative effect on performance in a Stroop test^29^.

However, all of these previous studies have used VR solely as a tool to display biophilic or non-biophilic content to the users. We found only two studies that offered navigation in the VR environment. The first one was the work of Chan et al.^52^ that showed navigating along a street in VR, which had vertical plants on its buildings’ walls, prevented a reduction in positive affect and showed an increase in stress measured by means of heart rate variability. Also, in our previous work^32^, we exposed participants to a virtual office environment with or without virtual plants and allowed them to navigate in each of these environments for some time before starting with the actual experimental task. As a result, we observed significantly higher cognitive performances in a working memory task when it was conducted in the virtual biophilic office. In comparison to the non-biophilic office environment, exposure to the biophilic virtual office resulted in higher positive affect and attentive coping, and lower anger and aggression.

### Cognitive processes of steering

Traditionally, motor and cognitive functions have been studied separately. Some researchers argue that motor function is a cognitive function^53^. Some others believe in a continuum that regulates the involvement of cognitive processes in the visual control of movement^54^. For example, Frens and Erkelens^55^ conducted an experiment that revealed that even basic pointing movements to visual targets are not entirely automatic, as their initial direction can be affected by other stimuli, such as auditory stimuli presented during the response period. These findings suggest that the process determining the direction of the hand movement is vulnerable to disruption, making it attention-dependent and involving cognitive functions^54^.

Path steering is a commonly used interaction task in graphical user interfaces. Like pointing, it involves users rapidly moving the input device from one place to another. However, unlike pointing, path steering is a more restricted movement that must be carried out within the confines of a designated path^56^. A frequently studied steering task is driving^57–59^. Numerous studies have shown the need for attention in steering while driving^60,61^. The SPIDER model of attention in driving explains the cognitive processes that are involved while driving. These include scanning, predicting, identifying, deciding on, and executing a response (SPIDER for short)^62^. These processes are dependent on limited capacities for attention and can be impaired by distraction^63^. In addition, if this attention-dependent task is not particularly interesting, attention fatigue can occur. Using a driving simulator, Thiffault and Bergeron^64^ investigated how roadside visual monotony influences driver fatigue and vigilance. They found that repetitive environments lead to increased steering movements and decreased alertness. Their findings suggest that disrupting visual monotony caused by natural elements such as trees, for instance, may help reduce driver boredom and fatigue.

Similar to driving in the real world or simulators, steering in 3D environments consists of several cognitive processes: perceiving the path, being able to see the next ‘step’ along the path in 3D, planning a motion towards that ‘step’, being able to move towards that ‘step’, and keeping the attention on the task to repeat that loop. Accordingly, this task involves 3D perception, 3D spatial cognition, 3D action, and attention. Therefore, the previously mentioned theories of ART and SRT may apply to path steering. This means that exposure to natural stimuli could have a positive impact on attentional capacities and cognitive functions required to perform steering tasks in real Life, as well as in 3D environments. Since we showed in our previous work^32^ that the presence of virtual plants in an immersive VR office environment can lead to improvements in both divergent and convergent cognitive tasks, we set out to examine whether the presence of virtual plants could also be beneficial for path steering. For this study, we used the same VR environment as in our previous study, and participants also had the chance to freely navigate in the virtual environment before starting with the actual experimental task.

### Steering in 3D virtual environments

According to Bowman et al.^65^, most interaction tasks in VR can be classified into three categories: (i) navigation, (ii) selection/manipulation, and (iii) system control. Typically, these spatial interaction tasks are combined, for example, when navigating to an object, selecting it, and manipulating it in space or selecting items from a 3D menu. There are some standard spatial interaction tasks to measure human performance in such tasks. One of the most well-known laws for modeling the user performance of pointing movements is Fitts’ law^66^. The model predicts the movement time of a pointing task as a function of the distance from source to target and the size of the target by:

1 $$\begin{aligned} T=a + b \cdot ID = a + b \cdot \log \left( \frac{L}{W} + 1 \right) \end{aligned}$$

where *a* and *b* are experimentally determined constants, *L* is the distance to the target, and *W* is the target width. The expression $\log \left( \frac{L}{W} + 1 \right)$is referred to as the Index of Difficulty (ID) of the task. Although Fitts’ law has been initially formulated for one-dimensional movements, it has been extended to model 2D and 3D pointing tasks, e.g^67–69^.,.

For path steering tasks, Accot and Zhai^70^ proposed the steering law in 1997. It suggests that a steering task can be broken down into a large number of segments, each of which can be treated as a goal-crossing task with the same index of ID. As a result, the total movement time can then be modeled by Fitts’ law, while the ID for the entire task is calculated by the sum of all the IDs of the segments. If the path width varies along the path, the general steering law is expressed by the following formula:

2 $$\begin{aligned} T=a+b \cdot ID = a + b \cdot \int _C \frac{dx}{W(x)} \end{aligned}$$

where a and b are empirically determined constants, C is a curved path, x is the elementary path length along C, and W(x) is the path width at path length x. This formula predicts the total time needed to perform the steering task. In case the path is straight with a length of L and a constant width of W, the model can be simplified as:

3 $$\begin{aligned} T=a+b \cdot ID = a + b \cdot \frac{L}{W} \end{aligned}$$

Based on this initial model for straight paths, researchers have suggested several extended models to account for steering behavior on a variety of paths. For instance, Liu et al.^34^ carried out two experiments aimed at assessing, validating, and modeling the influence of path curvature and orientation on steering performance within desktop-based 3D virtual environments. Their findings indicated that an increase in path curvature increases steering time, which is in line with previous work on Fitts’ law^71^. Their model can be expressed as:

4 $$\begin{aligned} logT= a + b \cdot log(\frac{L}{W}) + c\rho \end{aligned}$$

where a, b, and c are experimentally determined constants, L is the path length, W is the path width, and $\rho$ is the path curvature. In this study, we followed the experimental designs of the user studies proposed by Liu et al.^34^ to study the effects of biophilic design on steering performance in VR.

## Methods

### Virtual environments

**Fig. 1:**
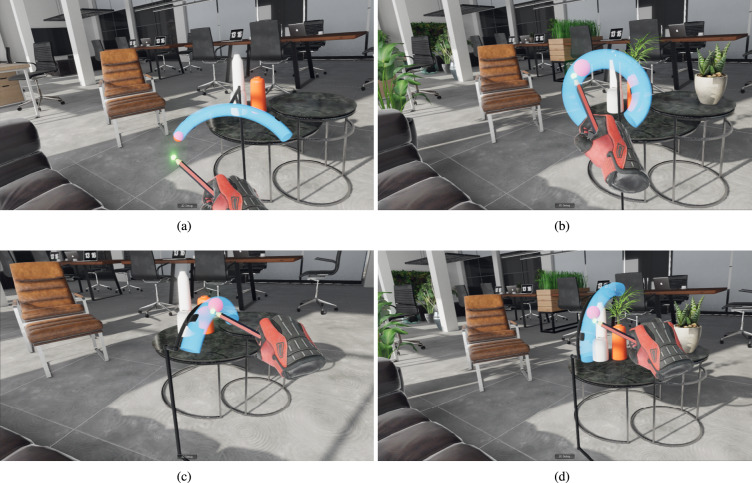
Sample steering tasks of (**a**) Scenario 1 in the non-biophilic office, (**b**) Scenario 1 in the biophilic office, (**c**) Scenario 2 in the non-biophilic office, and (**d**) Scenario 2 in the biophilic virtual office.

The virtual environments were a biophilic and a non-biophilic virtual office, as in our previous work^32^ built using the Unity3D game engine:

- **Non-biophilic** or the **no-plants** condition: in this environment, the virtual office was devoid of any plants (see Fig. 1(a) and (c)).
- **Biophilic** or the **plants** condition: in this environment, the same virtual office was enriched with 28 virtual 3D models of plants (see Fig. 1(b) and (d)).

The virtual environments were displayed within a Meta Quest 2 HMD with integrated headphones and controllers. The Meta Quest 2 was tethered with a Link cable to a Windows 10 PC with an i9-9900K CPU, RTX 2080 Ti GPU, ROG Strix Z390-F Gaming Mainboard, and 32 GB RAM.

### Steering tasks

As mentioned before, we replicated the work of Liu et al.^34^ to implement the steering task in VR. However, Liu and colleagues employed a desktop-based setup, and as a result, there was an offset between the physical input device and its virtual representation. Participants were seated at a table, using a stylus tracker and a head-tracked 6DOF stereoscopic display with a horizontal offset of 0.65 m and a vertical offset of 0.3 m between the motor and the visual space. In our study, though, we used a Meta Quest 2 HMD with inside-out tracking and projected the virtual position of the hands and pen directly onto the physical position.

Similar to the work by Liu and colleagues, we implemented two scenarios for evaluating steering tasks in VR. In the first scenario, besides the length and width of a path, varying curvatures and in the second scenario, varying spatial orientations were added to determine the steering time. The participants were asked to move a target ball through a semi-transparent tunnel by using a smaller cursor ball at the tip of a pen to push it to the other side. In both scenarios, the target ball was restricted by the inner walls of the tunnel, which set the tunnel’s diameter to its own. The width of the steering path is then defined by the target ball’s diameter plus two times the cursor ball’s radius. While Liu et al. showed a pen with the cursor ball on its tip as a visual representation of the real stylus, we used the HMD’s standard controllers and replaced them in VR with a model of a hand wearing a glove, and a pen on the respective side (see Fig. 1).

To push the target ball through the tunnel, the cursor ball had to touch the target ball and fully intersect the path. In our study, if it touched the wall of the tunnel while pushing the target ball, it turned red and passed through the ball. The tunnel only physically interacted with the target ball, and therefore, the cursor ball was able to pass through it at any time. Each task started as soon as the target ball left one end of the tunnel, and was completed when its center had reached the other end of the tunnel. The participant’s goal was to push it to the other end as fast as possible. As soon as one task was completed, the next one appeared. While there was no mention of breaks in the paper from Liu et al., we decided to include an interruption of the experiment roughly every 2 minutes for 10 seconds to ease the strain on participants’ muscles. We have also centered the position of the tunnels so that the range of movement was the same for left- and right-handed participants.

#### Scenario 1

The first scenario’s independent variables were the path’s length, width, and curvature. The tunnel was positioned in the xy-plane and started at the origin (center of virtual space). The lengths were chosen to be 0.24, 0.30, and 0.36 m, the radius of the cursor ball was fixed to 0.005 m, and the target ball’s radius varied between 0.01 and 0.015 m, resulting in path widths of 0.03 and 0.04 m. Liu et al.^34^ had five different curvatures: 0 (straight line), 4, 8, 12, and 16 $\hbox {m}^{-1}$. They also had three repetitions for each combination, resulting in 90 trials per participant. However, in our version of the scenario, the total trial count was reduced to 36 by excluding the curvatures 4 and 12 $\hbox {m}^{-1}$ and having only 2 repetitions per trial. The rationale was to avoid extending the duration of the scenario since these tasks were performed in two different virtual environments within the present study. Therefore, the paths in this scenario had the following properties:

- Length (L): 0.24, 0.30, and 0.36 m;
- Width (W): 0.03 and 0.04 m;
- Curvature ($$\rho$$): 0, 8, 16 $$\hbox {m}^{-1}$$

Figure 1 (a) and (b) show two sample steering tasks in the first scenario.

#### Scenario 2

Following the experimental setup of Liu et al.^34^, in the second scenario, the path length, width, and curvature were fixed (L = 0.24 m, W = 0.04 m, $\rho$) = 8 $\hbox {m}^{-1}$). The tunnel was first positioned in the xy-plane, with its left end (starting point) at the origin. The other end was then rotated around the y-axis and z-axis in varying combinations to reach different orientations. For the same reason as in Scenario 1, our version of the second scenario consisted of fewer trials than suggested by Liu et al. We implemented 8 different degrees of longitude (instead of 12) and 3 degrees of latitude (instead of 5) with 2 repetitions (instead of 3), which resulted in a total of 52 trials (instead of 186) per virtual environment for each participant. Thus, the path in this scenario had the following properties:

- Length (L): 0.24 m;
- Width (W): 0.04 m;
- Curvature ($$\rho$$): 8 $$\hbox {m}^{-1}$$
- Latitude ($$\alpha$$): $$-45^{\circ }$$, $$0^{\circ }$$, $$45^{\circ }$$
- Longitude ($$\beta$$): $$0^{\circ }$$, $$45^{\circ }$$, $$90^{\circ }$$, $$135^{\circ }$$, $$180^{\circ }$$, $$225^{\circ }$$, $$270^{\circ }$$, $$315^{\circ }$$

Figure 1 (c) and (d) show two sample steering tasks in the second scenario.

### Questionnaires

The study was conducted in the local language. Therefore, all task instructions and questionnaires were given in the local language. The questionnaires were completed on a separate laptop outside of VR with the following specifications: Windows 11 Home, Ryzen 5 5500U, 8 GB LPDDR4, 14” 1920x1200 IPS Display, and 256 GB SSD.

#### Simulator Sickness Questionnaire (SSQ)

We used the Simulator Sickness Questionnaire (SSQ) by Kennedy et al.^72^ to measure simulator sickness symptoms before VR exposure and after each virtual environment (i.e., biophilic and non-biophilic). The SSQ contains 16 items to describe physical symptoms that can occur because of exposure to VR. The items are rated on a four-point scale ranging from 0 (Not at all) to 3 (Very much). For this study, we analyzed the total SSQ score.

#### Igroup Presence Questionnaire (IPQ)

To measure the sense of presence in VR, we employed the Igroup Presence Questionnaire (IPQ)^73^. It consists of 14 items on a 7-point Likert scale ranging from 0 to 6 with different scale anchors, meaning that some items have general scale anchors (0 = Fully disagree to 6 = Fully agree) and some have more precise anchors (e.g., 0 = Not consistent and 6 = Very consistent). The questionnaire has four sub-scales: General Presence or the Sense of Being There, Spatial Presence, Involvement, and Experienced Realism. For the analysis, we also took into account the total sense of presence.

#### Perceived Restorativeness Scale (PRS)

This questionnaire measures the perceived restorativeness of an environment, which is relevant to query different aspects of ART^74–77^. The 26 items can be rated on a 7-point Likert scale from 1 (Not at all) to 7 (Completely). We analyzed the overall perceived restorativeness score.

### Procedure

We conducted a within-subject study, which was approved by the local ethics committee of the Center for Psycho-social Medicine at the University Hospital Hamburg-Eppendorf and was carried out in accordance with relevant guidelines and regulations. The study was conducted in a laboratory room at the University of Hamburg. The laboratory was exclusively reserved for this study so that no one except the experimenter and the participant was allowed to enter the room. This was done to control the environmental parameters, such as background noise. Upon arrival in the lab, participants were welcomed and presented with the participant information and the data protection declaration forms. They were informed that the study investigates how being in virtual offices affects spatial interaction tasks. Yet, they did not receive any information about our hypotheses and the fact that one of the office environments would contain plants, whereas the other would not. After signing the informed consent, participants filled out demographic and SSQ questionnaires and put on the Meta Quest 2 HMD. First, in a neutral virtual environment, the participants were asked to select their preferred handedness by touching either of the two cubes labeled as right-handed or left-handed. After that, they were randomly assigned to start with either the virtual office with plants or the one without them. At the start of each VR condition, they were greeted by an audio recording that gave them instructions on how to navigate through the virtual office environment using teleportation for a total of one minute (in addition to the duration of the given instructions). Afterward, they were instructed to sit on a sofa in the laboratory that corresponded to the position of a virtual sofa in the virtual office. The perspective of participants sitting on the sofa for both virtual environments and both scenarios is shown in Fig. 1. Another set of instructions followed, explaining the procedure and experimental tasks. During this time, teleportation was deactivated so that the participants could no longer leave their assigned location. In each scenario, as soon as one steering trial was completed, the next one appeared. Approximately every 2 minutes, we implemented a resting phase of 10 seconds to reduce the strain on participants’ muscles. As soon as the first scenario was completed, they had a 20-second resting phase before the second scenario was loaded. After both scenarios in the first virtual environment were complete, participants took off the HMD and filled out the questionnaires, i.e., SSQ, IPQ, and PRS. This procedure was repeated for the second virtual environment. A supplementary video to this paper depicts the procedure in both virtual office environments and the two scenarios. The video is provided in the original German language of the experiment. Nonetheless, we have added English subtitles that describe each step of the procedure. During the four VR conditions (two scenarios in each office), the steering time (how long it takes for the participants to push the target ball from one end of the tunnel to the other) was measured and saved to a file. Upon completing the tasks in both virtual environments, participants answered some open questions regarding their experience and whether they have plants in their Living or working spaces. At the end of the study, participants were compensated with course credits. The total study duration was approximately 35 minutes.

### Participants

Twenty-seven participants (44% women, 52% men, and 4% other) with an average age of 22.78 years (SD=2.83) took part in this study. They were mostly right-handed (74.07%). All of our participants had normal or corrected-to-normal vision. One of our participants reported having ocular dominance. No other vision disorders have been reported by our participants. All participants had used a VR headset before. Two individuals reported to have not have seen a 3D movie in a cinema before, and six did not have previous experience with 3D video games. On average, our participants reported spending 5.15 hours ($SD=7.41$) per week playing video games.

### Statistical analysis

**Fig. 2:**
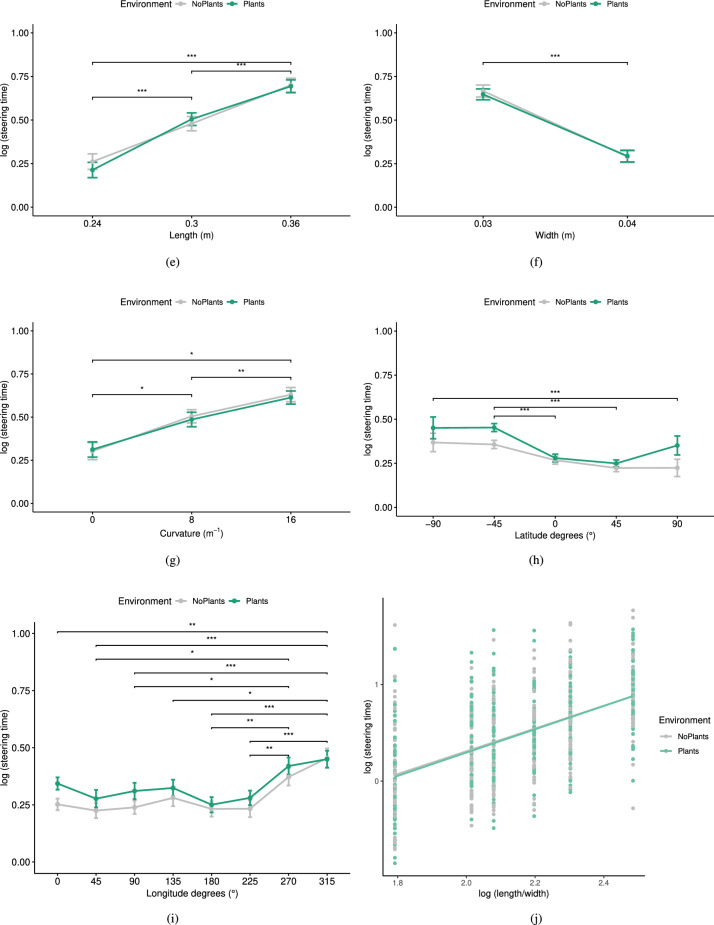
$log(T_{steering})$ in different virtual environments across different (**a**) lengths, (**b**) widths, (**c**) curvatures, (**d**) latitudes, and (**e**) longitudes. The classical steering time vs. ID = log(L/W) in both environments is shown in (**f**), where the green line shows the model fitting for the virtual office with plants and the gray line shows the fitting line for the virtual office without plants.

We employed the Grubbs test^78^ to search for and remove outliers in the steering time data. In addition, six single data points had to be deleted due to system glitches and errors. The normality of the data was assessed by running the Shapiro-Wilk test. To perform frequentist repeated-measures ANOVAs (RM ANOVAs), we tested the sphericity assumption using Mauchly’s test of sphericity. If the assumption was violated, we corrected the degrees of freedom using Greenhouse-Geisser estimates of sphericity. As an effect size, we report the partial eta squared ($\eta _p^2$), whereby a value of.01 was considered a small effect,.06 a medium effect, and.14 a large effect^79^. If the RM ANOVA was significant, we conducted pairwise comparisons and corrected the p-values using the Bonferroni method. In Fig. 2, asterisks represent Bonferroni adjusted p-values (* for $p <.05$, ** for $p <.01$, and *** for $p <.001$) of post-hoc tests. To analyze the responses to IPQ and PRS questionnaires, we performed paired t-tests and reported Cohen’s |*d*| as the effect size. It is commonly interpreted as small ($|d| =.2$), medium ($|d| =.5$), and large ($|d| =.8$) effects^79^. The significance level was set for all tests at $\alpha =.05$.

## Results

### Scenario 1

Thirty-two data points from the steering time recordings ($1.66\%$ of all steering time data points in Scenario 1) were detected as outliers and excluded from the analysis. The logarithm transformation was employed to bring the steering time distribution closer to a normal one. The Kolmogorov-Smirnov test ($D1 =.04, p=.26$) verified this transformation, and the Shapiro-Wilk test confirmed its normality afterward ($p=.15$).

We performed a 2 (Environment) x 3 (Length) x 2 (Width) x 3 (Curvatures) RM ANOVA on the transformed $T_{steering}$. The means (M) and standard deviations (SDs) of $T_{steering}$ for each of these factors can be seen in Table 1. The results of RM ANOVA showed a significant main effect of Length ($F(1.88, 15) = 106.12, p <.001, \eta _{p}^{2}=.93$), Width ($F(1,8) = 177.2, p <.001, \eta _{p}^{2}=.96$), and Curvature ($F(1.21, 9.68) = 14.07, p =.003, \eta _{p}^{2}=.64$). However, no significant main effect of the Environment ($F(1,8) = 0.00, p =.95, \eta _{p}^{2} <.001$) and no significant interaction effects were observed. Bonferroni adjusted comparisons showed that the steering time was significantly less for the paths with a length of 0.24m compared to the ones with a length of 0.3m ($p<.001$) and 0.36m ($p<.001$) and for the paths with a length of 0.3m compared to the ones with a length of 0.36m ($p<.001$). The steering time was also significantly lower for the paths with a width of 0.04m ($p<.001$) compared to the ones with a width of 0.03. For the straight paths (i.e., curvature = 0), the steering time was significantly lower compared to the paths with a curvature of 8 $m^{-1}$ ($p=.04$) and 16 $m^{-1}$ ($p=.01$). The steering time was also significantly higher for the paths with the curvature of 16 $m^{-1}$ compared to the ones with the curvature of 8 $m^{-1}$ ($p=.02$). See Fig. 2 (a-c) for a visualization of these results.

In addition, we examined whether a linear relationship between $log(T_{steering})$ and $log(\frac{L}{W})$ can be observed for each path curvature $\rho$ as presented in Equation 4. For the biophilic office, the adjusted $R^{2}$ of 0.38 suggests that approximately 38.4% of the variance in the $log(T_{steering})$ can be explained by the model. Thus, the model does account for a substantial portion of the variance, though additional factors may also play a role in predicting the steering time. The results of the regression analysis are summarized in the Table 2. As can be seen, the coefficient b of 1.21 indicates a positive relationship between $log(\frac{L}{W})$ and $log(T_{steering})$. The same can be applied to the coefficient c of 0.15 value, which shows that $\rho$ has a positive influence on the steering time. These effects are also significant, as the confidence intervals do not include zero. Similar results can be observed for the non-biophilic office, as the adjusted $R^{2}$ was 0.36 and both $log(\frac{L}{W})$ and $\rho$ have positive influences on $log(T_{steering})$ (see Table 2 and Fig. 2f).

**Table 1:** Mean (Standard Deviation) of $T_{steering}$ for each independent variable in Scenario 1.

**Environment**	M (SD)	**Length**	M (SD)	**Width**	M (SD)	**Curvatures**	M (SD)
NoPlants	.48(.49)	0.24m	.24(.48)	0.03m	.66(.44)	0$m^{-1}$	.31(.51)
Plants	.47(.47)	0.3m	.49(.42)	0.04m	.29(.45)	8$m^{-1}$	.5(.44)
		0.36m	.7(.42)			16$m^{-1}$	.62(.43)

**Table 2:** Parameter estimates of Equation 4 fitting on the total steering time in Scenario 1.

Environment	Coef.	Estimate	t value	$P(>|t|)$	[95% conf. interval]
NoPlants	a	−2.4	−11.33	$<.001$	[−2.82,−1.98]
	b	1.18	12.47	$<.001$	[.1, 1.37]
	c	.17	6.59	$<.001$	[.12,.22]
Plants	a	−2.42	−12.29	$<.001$	[−2.8,−2.03]
	b	1.21	13.63	$<.001$	[1.03, 1.38]
	c	.15	6.33	$<.001$	[.1,.2]

**Table 3:** Mean (Standard Deviation) of $T_{steering}$ for each independent variable in Scenario 2.

**Data set**	**Environment**	M (SD)	**Latitude**	M (SD)	**Longitude**	M (SD)
Latitudes [$-45^{\circ }$,$0^{\circ }$,$45^{\circ }$]	NoPlants	.28(.38)	$-45^{\circ }$	.4(.39)	$0^{\circ }$	.29(.39)
	Plants	.34(.38)	$0^{\circ }$	.28(.39)	$45^{\circ }$	.28(.38)
			$45^{\circ }$	.25(.35)	$90^{\circ }$	.28(.35)
					$135^{\circ }$	.32(.35)
					$180^{\circ }$	.24(.35)
					$225^{\circ }$	.26(.36)
					$270^{\circ }$	.39(.39)
					$315^{\circ }$	.44(.42)
Pole Latitudes [$-90^{\circ }$and$90^{\circ }$]	NoPlants	.32(.35)	$-90^{\circ }$	.44(.36)	$0^{\circ }$	.36(.35)
	Plants	.39(.35)	$90^{\circ }$	.28(.32)	$0^{\circ }$	.36(.35)

**Table 4:** Parameter estimates of Equation 5 fitting on the total steering time in Scenario 2 for latitudes [$-45^{\circ }$, $0^{\circ }$, $45^{\circ }$] and parameter estimates of Equation 6 fitting on the total steering time in Scenario 2 for pole latitudes [$-90^{\circ }$ and $90^{\circ }$].

Data set	Environment	Coef.	Estimate	t value	$P(>|t|)$	[95% conf. interval]
Latitudes [$-45^{\circ }$, $0^{\circ }$, $45^{\circ }$]	NoPlants	a	.38	7.15	$<.001$	[.28,.49]
		b	-.07	−4.31	$<.001$	[-.096, -.04]
		c	.02	3.99	$<.001$	[.01,.03]
	Plants	a	.55	10.43	$<.001$	[.45,.65]
		b	-.09	−6.11	$<.001$	[-.12, -.06]
		c	.01	2.71	$<.01$	[.004,.03]
Pole Latitudes [$-90^{\circ }$ and $90^{\circ }$]	NoPlants	a	.45	6.93	$<.001$	[.32,.58]
		b	-.04	−2.36	.02	[-.08, -.007]
	Plants	a	.52	7.92	$<.001$	[.39,.65]
		b	-.04	−2.25	.03	[-.08, -.005]

### Scenario 2

The analysis of the data from Scenario 2 was done separately for the set of the three “middle” latitudes ($-45^{\circ }$, $0^{\circ }$, $45^{\circ }$) and the pole latitudes of $-90^{\circ }$ and $90^{\circ }$ because there was only one longitude for these two pole latitudes. Twenty data points from steering time recordings for the latitudes between $-45^{\circ }$ and $45^{\circ }$ ($1.1\%$ of all steering time data points for these latitudes) and five data points from the steering times for the two poles ($2.84\%$ of all steering time data points for the $-90^{\circ }$ and $90^{\circ }$ latitudes) were detected as outliers using the Grubbs test and were removed for the analysis. Similar to Scenario 1, the steering times for the two data sets of Scenario 2 (i.e., for the latitudes between $-45^{\circ }$ and $45^{\circ }$ and the pole latitudes) were also transformed logarithmically to observe an approximately normal distribution. The means and standard deviations of $T_{steering}$ for each independent variable can be seen in Table 3 for both data sets. Also, Fig. 2 (d-e) shows a visualization of the results.

The results of a 2 (Environment: plants, no-plants) $\times$ 3 (Latitude: $-45^{\circ }$, $0^{\circ }$, $45^{\circ }$) $\times$ 8 (Longitude: $0^{\circ }$, $45^{\circ }$, $90^{\circ }$, $135^{\circ }$, $180^{\circ }$, $225^{\circ }$, $270^{\circ }$, $315^{\circ }$) RM ANOVA on $log(T_{steering})$ showed a significant effect of Latitude ($F(1.72, 31.02) = 24.12, p <.001, \eta _{p}^{2}=.57$) and Longitude ($F(3.39, 61.03) = 3.81, p =.01, \eta _{p}^{2}=.18$) but no significant effect of the Environment ($F(1, 18) = 2.13, p =.16, \eta _{p}^{2}=.11$). Also, no significant interaction effect could be observed. Pairwise comparisons with Bonferroni adjustment showed that steering at the latitude of $-45^{\circ }$ was harder and took significantly more time for participants compared to steering at the latitudes of $0^{\circ }$($p<.0001$) and $45^{\circ }$ ($p<.0001$). Pairwise comparisons for Longitude showed that the longitude of $315^{\circ }$ took the longest time for steering which was significantly more than the longitude of $0^{\circ }$ ($p=.002$), $45^{\circ }$ ($p<.001$), $90^{\circ }$ ($p<.001$), $135^{\circ }$ ($p=.015$), $180^{\circ }$ ($p<.001$), and $225^{\circ }$ ($p<.001$). After that, the longitude of $270^{\circ }$ took significantly more time for steering compared to the longitude of $45^{\circ }$ ($p=.044$), $90^{\circ }$ ($p=.016$), $180^{\circ }$ ($p=.002$), and $225^{\circ }$ ($p=.002$).

Furthermore, we examined whether $log(T_{steering})$ has a linear dependence on latitude ($\alpha$) and longitude ($\beta$) as shown in Equation 5, in which a, b, and c are experimentally determined constants. For the non-biophilic office, the adjusted $R^{2}$ of.036 shows that approximately 3.6% of the variance in $log(T_{steering})$ is explained by the model. The results of the regression analysis are summarized in Table 4. The coefficient b of -.07 indicates a negative relationship between latitude and $log(T_{steering})$. For longitude, the coefficient c of.02 shows a positive effect on the steering time. These effects are significant, as the confidence intervals do not include zero. For the biophilic office, the adjusted $R^{2}$ of.047 suggests that approximately 4.7% of the variance in $log(T_{steering})$ is explained by latitude and longitude combined. There is a significant negative relationship between the latitude and the steering time, meaning that as latitude increases, the steering time tends to decrease (estimated coefficient = -.09). For the longitude, a significant positive relationship can be observed, as with an increase in the longitude, steering time tends to increase slightly (estimated coefficient =.01).

5 $$\begin{aligned} log(T_{steering})= a + b \cdot \alpha + c \cdot \beta \end{aligned}$$

For steering with the pole latitudes, we ran a 2 (Environment: plants, no-plants) $\times$ 2 (Latitude: $-90^{\circ }$, $90^{\circ }$) RM ANOVA which showed a significant effect of Latitude ($F(1, 21) = 9.78, p =.005, \eta _{p}^{2}=.32$) but no significant effect of the Environment ($F(1, 21) = 1.7, p =.21, \eta _{p}^{2}=.08$) or its interaction with the Latitude. A paired t-test with Bonferroni adjustment revealed that steering at the latitude of $-90^{\circ }$ ($p=.001$) took significantly more time for participants compared to steering at the latitude of $90^{\circ }$.

Also, for this data set, we examined whether there is a linear relationship between $log(T_{steering})$ and latitude ($\alpha$) as shown in Equation 6, in which a and b are experimentally determined constants. Table 4 includes the regression analysis for this subset, too. For the non-biophilic office, the adjusted $R^{2}$ of.061 shows that approximately 6.1% of the variance in $log(T_{steering})$ is explained by the model. Additionally, a significant negative effect (estimated coefficient = -.04) can be seen between the latitude and steering time. Similar effects can be seen for the biophilic office, where there is a significant negative effect of the latitude on the steering time (estimated coefficient = -.04), and the $R^{2}$ of.056 suggests that 5.6% of the variance in the steering time can be explained by this model.

6 $$\begin{aligned} log(T_{steering})= a + b \cdot \alpha \end{aligned}$$

All in all, H1 could be supported, but H2 had to be rejected by our findings in both Scenario 1 and Scenario 2.

### Questionnaires

Table 5 shows the means and standard deviations of all questionnaire values.

**Table 5:** Mean (Standard Deviation) of all questionnaires.

Questionnaire	Pre	(Post) NoPlants	(Post) Plants
**SSQ**	12.19(14.51)	17.18(19.58)	15.65(17.91)
**IPQ**			
Total sense of presence		3.14(.64)	3.27(.59)
General sense of presence		4.56(1.42)	4.63(1.15)
Spatial presence		3.59(.87)	3.67(.86)
Involvement		2.54(1.02)	2.74(.81)
Experienced realism		2.83(.91)	2.97(.81)
**PRS**		4.76(1.2)	5.02(1.09)

#### Simulator Sickness (SSQ)

**Fig. 3:**
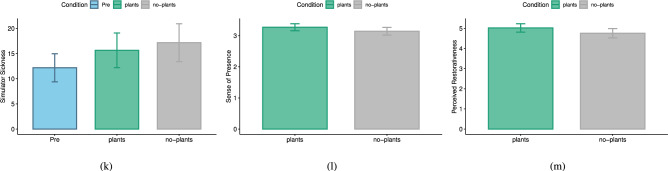
The results of questionnaires: (**a**) simulator sickness (SSQ), (**b**) Igroup presence questionnaire (IPQ), and (**c**) perceived restorativeness scale (PRS).

We ran a repeated measures ANOVA on SSQ measurements (see Fig. 3a) and did not find a significant difference between the SSQ measurements before starting with the VR study and after the plants and no-plants conditions $(F(1.81, 47.09) = 1.03, p =.36, \eta _{p}^{2} =.038)$. Therefore, H3 cannot be supported by these results.

#### Igroup Presence Questionnaire (IPQ)

The results of the paired t-tests did not show any significant differences between the plants and no-plants conditions in the total sense of presence ($t(26)=.95, p=.35, |d|=.18$), the general sense of presence ($t(26)=.18, p=.86, |d|=.04$), spatial presence ($t(26)=.36, p=.72, |d|=.07$), involvement ($t(26)=.91, p=.37, |d|=.18$), experienced realism ($t(26)=.61, p=.55, |d|=.12$). Thus, H4 has to be rejected. Figure 3bdepicts the total IPQ score for both virtual environments.

#### Perceived Restorativeness Scale (PRS)

No significant differences between the total PRS score (see Fig. 3c) of the virtual environment with plants and no-plants could be found ($t(26)=.73, p=.47, |d|=.14$). Accordingly, H5 has to be rejected.

#### Further questions

After completing the experimental tasks and questionnaires in both virtual offices, participants answered some open questions regarding their experience and whether they have plants in their living or working spaces. The first question was formulated as “Do you have any idea what the difference between the two offices was? If so, please describe it briefly.” In response to this question, only 10 participants (37%) could name plants as the difference. The rest (63%) did not notice any Change or guessed incorrect differences, while a few noticed a difference but were unable to state what it exactly was. The next question probed whether participants had plants at home and at their workplace. As a result, 51.85% stated that they had plants at home, but more than half of the participants (55.56%) stated that they did not have plants at their workplace. In case they have indoor plants at their workplace, we further asked whether they think that the plants influence their abilities. Out of 11 responses that we received, one person stated “There are not that many house plants at my workplace. The majority of them are made of plastic. Accordingly, I do not think they have too much of an effect on my abilities.” and another person wrote that “No, at most it distracts me because I think about whether I have already watered it!”. The rest of the participants clearly wrote that they think plants have benefits for them, such as “calming and concentration”, “the more natural and friendly environment makes me feel better”, “positive, more power and less feeling of exertion, happier overall”, “better mood, more motivation, higher performance, better well-being”, and “better air”.

## Discussion

In this study, we combined two previous studies. The first one was our previous work^32^, which showed that the presence of virtual plants in a virtual office environment leads to higher performance in a digit span and a creativity test compared to the same environment without plants. To study whether our previous results extend to a motor-cognitive task, we applied the steering tasks presented by Liu et al.^34^ in a virtual office with or without virtual plants.

We could replicate the findings of the steering task in VR proposed by Liu et al.^34^, as the steering time in our study follows the extension of the steering law proposed in their study. This steering law considers not only the length and width of the steering path as the index of difficulty, but also includes path curvature as a factor in affecting the time needed for steering through the path. We also found significant effects of path latitude and longitude on steering time. However, the small estimated coefficients suggest these variables have only a modest influence on steering time. Also, the small $R^{2}$ values indicate that most of the variation in steering times is influenced by factors not included in our models. Thus, additional factors may play a role in predicting the steering time. Factors such as task speed^80^ or frame rates^81^ could probably provide a more accurate model for path steering in VR.

However, our results did not extend our previous findings^32^ to steering in VR. That is, we could not find an effect of the presence or absence of virtual plants on steering time. Our study participants performed all steering tasks in a similar amount of time in both virtual offices. Furthermore, we could not replicate our previous findings regarding the simulator sickness, the sense of presence, and the perceived restorativeness qualities of the virtual environment. There are some differences between the administration of these measures in our previous study compared to the present one.

The first regards the SSQ, which our previous study measured only before two post-exposure measurements and after the entire study. Therefore, no information about the SSQ after each virtual environment was available. The total SSQ score in our previous study increased significantly from pre- ($M=13.62, SD=14.09$) to post-measurement ($M=23.02, SD=16.67$). Our present study did not lead to increased simulator sickness symptoms, as the SSQ score in our present study before exposure ($M=12.19, SD=14.51$) was comparable to the previously reported pre-SSQ score, but the measurements after the plants ($M=15.65, SD=17.91$) and no-plants ($M=17.18, SD=19.58$) conditions were generally lower than the previously reported post-SSQ measurement. Probable reasons could be the length of the study, which was longer in the previous study (approximately 50 minutes compared to 35 minutes in the present study), and the experimental tasks, which were different in both studies.

The sense of presence was measured only using the first item of IPQ (i.e., the general sense of presence) in our previous work^32^, which was significantly higher for the biophilic office ($M=5.59, SD=.82$) compared to the non-biophilic one ($M=5.23, SD=.87$). However, neither the total sense of presence nor any sub-scales of IPQ, including the general sense of presence, were significantly different in our present study. The sense of presence for both conditions (plants: $M=4.63, SD=1.15$, no-plants: $M=4.56, SD=1.42$) was also rated lower in our present study compared to our previous work.

The perceived restorativeness was also significantly higher in our previous study for the biophilic office compared to the non-biophilic environment. Although our measurements for these two environments (plants: $M=5.02, SD=1.09$ and no-plants: $M=4.76, SD=1.2$) are comparable to the previous data reported, we could not detect a significant difference between them.

Multiple reasons could have contributed to this discrepancy between the findings of our present study and the previous study, especially regarding the effects of biophilic design. The first one is regarding the presentation of the experimental task. While our steering task was presented visually, the cognitive tasks in our previous study were administered in the auditory domain. The visual presentation of our task might have blocked (or at least reduced) the participants’ perception of the virtual plants and thereby hindered their positive effects on all our study measures. Previous research has compared different stimulus modalities and has demonstrated that auditory and visual tasks elicit different responses and potentially, different underlying cognitive processes^82^.

Moreover, most of our participants (63%) did not notice the virtual plants in one of the environments and could not name any correct differences between the two virtual office environments. We intentionally decided not to reveal the main hypothesis of our study to the participants at the beginning of the study to prevent any biased performance. However, the fact that the plants were not noticed could be a potential reason for not benefiting from them.

Another reason could be the duration of the two studies, which were different. The participants in our previous study had a longer time of exposure to the virtual plants. Although studies such as Jun et al.^50^ show that even a 2-minute exposure can lead to beneficial effects of virtual biophilic design, the duration of the VR nature exposure is still debatable^83^. Therefore, it is still not clearly understood how long virtual nature exposures should be, especially in interactive VR applications, where participants have to perform visually administered tasks.

A Limitation of our study is that the observed effects were assessed following a single exposure. As a result, we cannot make any conclusions regarding the effects of repeated exposures or the long-term effects of virtual biophilic office environments. Another Limitation of our study is its relatively young sample with an average age of 22.78 years from a WEIRD (Western, Educated, Industrialized, Rich, and Democratic) population^36^. As such, the results cannot be generalized to other user groups. Future research should include a more diverse population for testing the effects of virtual biophilic design. A further limitation of our study is the lack of systematic control over the physical environment. Although we attempted to keep factors such as room temperature and background noise constant for all participants, we do not have any measures of the quality of this control, since we did not systematically record temperature or noise levels. Future studies could increase the reliability of the outcomes by accounting for these factors, as they can potentially affect cognitive performance.

Moreover, our experimental task was a spatial interaction task, and we could not find any other study in this field that has compared the effects of biophilic design on such tasks. In one of our previous studies, we compared performing redirected walking tasks while simultaneously performing a visually-administered 2-back test in virtual nature and urban environments^84^. However, similar to the present study, this previous study also could not observe an effect of the virtual environment on our previously employed study measures, including the above-mentioned task performances as well as the perceived restorativeness, sense of presence, simulator sickness, and psychological well-being. Our results are in Line with these previous findings concerning the subjective evaluations of the virtual environments. It is worth noting that the previously administered 2-back test was also presented visually to the participants, which might have contributed to the observed results (as we discussed above).

Although we could not observe positive effects of virtual plants on steering tasks in our experimental setup, the potential positive effects of biophilic design on motor-cognitive tasks in the built environment should not be neglected in future research. Various tasks in the built environments demand ongoing attention, especially those that are repetitive and monotonous. Tasks involving steering or manual hand movements in the built environment include, for example, operating machinery, assembling or installing components, and driving carts and trolleys in warehouses or large facilities. Biophilic design may facilitate the restoration of attention and recovery from stress, particularly in monotonous settings, thereby maintaining safety, accuracy, and efficiency.

## Conclusion

In this study, we set out to examine whether the presence of virtual plants in an immersive VR office environment could be beneficial for path steering. For this purpose, we combined and replicated two previous studies. The first one was our previous work^32^, which showed that the presence of virtual plants can lead to improvements in both divergent and convergent cognitive tasks. For this study, we used the same VR environment as in our previous study. The second study that was replicated was the work of Liu et al.^34^, which presented a method for modeling path steering in 3D VR environments.

Our results did not find an effect of the presence of virtual plants on our study measures, including steering performance, sense of presence, and perceived restorativeness. However, our results support the work of Liu et al.^34^, as the steering time in our study follows the extension of the steering law proposed in their study. This steering law considers not only the length and width of the steering path as the index of difficulty, but also includes path curvature as a factor in affecting the time needed for steering through the path. We also found significant effects of path latitude and longitude on steering time. However, our results suggest that these variables have only a modest influence on steering time, and additional factors such as task speed^80^ or frame rates^81^ could probably provide a more accurate model for path steering in VR. Thus, our results challenge the findings of previous research, which demonstrated the positive effects of biophilic design in VR on cognitive processes, at least for visuo-motor tasks and subjective sense of presence in those environments. Therefore, more research is needed to facilitate an understanding of whether and how biophilic designs in VR, particularly in interactive VR systems, can contribute to improving users’ mental processes in these immersive environments.

## Supplementary Information

Below is the link to the electronic supplementary material.

Supplementary Material 1

## Data Availability

The datasets used and analyzed during the current study will be available from the corresponding author upon reasonable request.
